# Stigmatizing imagery for substance use disorders: a qualitative exploration

**DOI:** 10.1186/s40352-023-00229-6

**Published:** 2023-07-04

**Authors:** Jessica Hulsey, Kayla Zawislak, Ginnie Sawyer-Morris, Valerie Earnshaw

**Affiliations:** 1 Addiction Policy Forum, 4701 Sangamore Road, Suite 100N, Bethesda, MD 20816 USA; 2 University of Delaware, Newark, DE 19716 USA

## Abstract

**Background:**

Stigma is a significant barrier to the treatment of individuals with substance use disorders. While prior efforts have been made to change stigmatizing language to refer to individuals with substance use disorders (SUD), little is known about the effects of stigmatizing imagery. There is a need for complementary qualitative research to identify both stigmatizing and non-stigmatizing imagery in the field of SUD.

**Methods:**

This study used qualitative methods to identify stigmatizing and non-stigmatizing imagery for SUD and explore the reactions of people with lived experience with SUD to SUD-related imagery. We conducted focus groups and brief semi-structured qualitative interviews with 14 individuals in recovery from a range of SUD.

**Results:**

Participants identified images of substance use and criminal justice contact that are negative or stigmatizing, along with alternative images that were endorsed for use. The unanticipated concept of imagery-induced triggering and cue reactivity emerged in the interviews, along with an emphasis on diversity in race/ethnicity, gender, and age for representations of both patients and clinicians in all imagery.

**Conclusions:**

The findings can be helpful in informing imagery that can depict addiction, individuals with SUD, and individuals involved in the justice system for various fields from research to media, public health, and community-based programming. Based on qualitative feedback from patients on triggering effects and reactivity to visual cues, it is never appropriate to use drug use and drug paraphernalia imagery to depict substance use or misuse or pictures of people in cages.

## Background

Individuals with a substance use disorder frequently experience stigma, which includes prejudice, stereotypes, and discriminatory treatment. Stigma results from a social process in which certain marks are constructed as indicators of tarnished character. These marks are used to justify discrimination and power loss of people with that characteristic, such as addiction (Earnshaw, 2020). The effects of stigma can hamper treatment, recovery, and reintegration outcomes. Individuals with substance use disorder (SUD) who experience stigma are more likely to continue engaging in substance use potentially as a way to cope with the stigma they are facing (Tsai et al., 2019), manifest greater delayed treatment access and higher rates of dropout (Corrigan et al., 2006), and show reduced help-seeking behaviors (Stangl et al., 2019). The *National Survey on Drug Use and Health* found that 22.7 percent of individuals in need of treatment reported that stigma kept them from pursuing addiction treatment (SAMHSA, 2015). Both imagery and language are powerful tools of communication and mechanisms of behavior change (Daundasekara et al., 2019); they serve as a means to identify, label, and alienate stigmatized groups (Ashford et al., 2018). Although some research has identified stigmatizing language, little work to date has explored stigmatizing imagery. Research that identifies stigmatizing imagery can inform recommendations for best practices in communicating about substance use in the media, research publications, and other institutions.

Studies on stigmatizing language in the context of substance use have found that stigmatizing descriptions of people with SUDs (e.g., “addict” as opposed to “person with a substance use disorder”) can lead to more negative affect toward those individuals (Goodyear et al., 2018), increased implicit bias (Ashford et al. 2018), greater attributions of responsibility (Kelly & Westerhoff, 2010), and increased desire for punitive action (Kelly & Westerhoff, 2010). Recently, Ledford et al., (2021) investigated whether stigma message features exert direct effects on stigma-related outcomes. They found that stigmatizing language that included stigma message features such as marks (e.g., “Alex appears unkempt”) and labels (e.g., “opioid addict”) was linked to greater perceptions of dangerousness and threat, as well as an increased desire for behavioral regulation (i.e., the desired isolation and intervention of the stigmatized group) and social distance (Ledford et al., 2021). Interventions to reduce the stigma associated with substance use disorders include language modifications – replacing terms that reinforce discrimination and stereotypes like “substance abuser” and “addict” with person-first language, such as an individual with a substance use disorder.

Institutions and various systems have begun to change the language around addiction. In 2015, the International Society of Addiction Journal Editors released a statement and guidelines for addiction terminology that “recommends against the use of terminology that can stigmatize people who use alcohol, drugs, other addictive substances, or who have an addictive behavior.” The Associated Press in 2017 updated its stylebook with guidance to avoid addiction terms like abuse, addict, and abuser. The American Medical Association (2021) released guidance on stigmatizing language, which states that “certain words can make patients feel unsafe or excluded or impose limitations, which can affect their well-being.” Although changing language alone may not eradicate stigma, it can pave the way for more intensive stigma interventions.

The majority of research and advocacy to date has focused on stigmatizing language; however, language is not the only means by which stigma is perpetuated within communications about substance use. Stigmatizing imagery (i.e., images that implicitly or explicitly reinforce stereotypes and prejudice towards stigmatized individuals) depicting drugs or substance use also has the potential to foster SUD stigma. Similar to language identified as stigmatizing, these images may impact the way members of the general population think, feel, and respond to people with SUD. Stigmatizing imagery may additionally negatively impact people with SUD, leading them to perceive, anticipate, or internalize stigma. Given the potential power of imagery to activate strong affective responses, images of drugs and substance use may additionally trigger trauma or relapse responses for those with lived experience.

Besides promoting stigma associated with substance use, stigmatizing communications may promote intersectional SUD-related stigma (i.e., stigma associated with other statuses experienced by people with SUD). This is particularly important to consider in the context of imagery given that visual communication can often convey more complex messages than written communication (Reed, 2013). For example, images depicting Black, Hispanic, LGBTQ + , or justice-involved individuals using drugs may promote racism, xenophobia, homophobia, or criminal justice stigma alongside SUD stigma—various forms of “othering”. Of note, many people with SUD have a history of criminal justice involvement. Stigma associated with SUD and criminal justice involvement is associated with greater psychological distress, decreased self-esteem (Turney et al., 2013), and greater social isolation (Moore and Tangney, 2017).

Both imagery and language are used to document the opioid epidemic and while previous research has recommended against imagery that “marks” individuals with SUD (Ledford et al., 2021), more evidence is needed to understand what constitutes non-stigmatizing imagery in substance use contexts. Our study seeks to address this gap by exploring responses from individuals with lived experience to imagery used to represent SUD and criminal justice involvement. Modifying imagery and language used to describe and represent individuals with a SUD or criminal justice involvement is an intervention that researchers, institutions, and other perceivers can implement.

## Methods

In July 2020, the research team worked with key stakeholders engaged to provide recommendations on the use of SUD-related imagery in communications about SUD to the Justice Community Opioid Innovation Network (JCOIN). Finding little research on SUD-related stigmatizing imagery in the empirical or gray literature, investigators conducted a qualitative study following Rapid Qualitative Inquiry (RQI) methods with people with lived experience with SUD. Goals of the study were to explore participants’ responses to SUD-related images and classify SUD-related images as stigmatizing or non-stigmatizing. Analyses focused on identifying themes to inform recommendations surrounding SUD-related imagery.

### Recruitment

The research team recruited participants through email and phone communication with Addiction Policy Forum’s (APF) network of individuals with lived experience, including individuals who receive clinical services from APF, participate in APF’s recovery programming, or serve on advisory boards. Individuals were eligible to participate if they were 18 years or older, spoke English, and identified as either a person in recovery or as a person with an active SUD. Justice-involved individuals were purposively sampled given intersections of substance use and criminal-justice stigmas. Interested participants were referred to the research team to confirm eligibility, obtain additional details if necessary (e.g., stage of recovery), and schedule the focus group. Fourteen individuals who identify as having a substance use disorder in their lifetime participated in semi-structured focus groups or individual interviews conducted virtually using Zoom in December 2020 and June 2022. Nine individuals participated in three different focus groups. Five individuals participated in individual interviews to accommodate participants’ schedules. The research team recorded and transcribed all interviews. Once the study reached 14 interviews, interviews were concluded as narratives reached saturation. Participants provided informed consent prior to data collection activities and were given a $20 gift card upon completion. Approval for this study was granted by the Advarra Institutional Review Board (IRB no. Pro00056499).

### Qualitative procedures

Investigators developed a semi-structured interview guide to understand how individuals with SUD and justice involvement perceive imagery frequently utilized to depict them. The team compiled depictions of addiction from the top ten print and web news outlets published in the three years prior to data collection. Images were identified for six distinct categories of representation of individuals with substance use disorder: 1) treatment and patient services, 2) types of substance use disorder, 3) law enforcement contact, 4) court and pretrial proceedings, 5) carceral settings, and 6) reentry and community supervision. Examples of addiction-related and criminal justice imagery were then collected from the sources along with representative stock photo images sampled from commonly used websites and stock photography repositories. In total, 46 images were used alongside the interview guide to elicit participant feedback and discussion. Participants categorized each image as endorsed (i.e., “do use”) or not endorsed (i.e., “do not use”) for public usage followed by probing questions to help record detailed responses and feedback for each image. For example, the study asked all participants the question, “Do you recommend the media and organizations do use this image, or don’t use this image?” and the probing question on stigma, “Does this image reinforce negative or unfair beliefs about individuals with substance use disorders?” Interviewers asked additional probing questions related to the study aims.

### Analysis

The RQI approach facilitates quickly developing preliminary understanding of participant responses to a range of imagery related to addiction (Beebe, 2014). The RQI was team-based and involved rapid coding, which enabled the research team to disseminate the findings quickly, share patient feedback on stigmatizing imagery, and provide recommendations to the practitioner and research communities. The RQI focused on categorizing images as endorsed or not endorsed, identifying main themes, and generating high-level recommendations surrounding use of SUD-related images.

The virtual focus group format allowed for the research team and participants to collaborate and validate findings in real time. Participants’ categorized each image as do or do not use during the focus group interview, and the coding was validated by each participant before progressing to the next image. The process was iterative and allowed for investigators to add to and modify the interview protocol and materials in real time and in collaboration with participants. For example, none of the initial images provided by the study team to represent types of SUD (opioid use disorder, alcohol use disorder) were endorsed by participants; all initial images were considered stigmatizing. Members of the team therefore searched the internet for new images to add to the interview protocol. New images were shared until participants endorsed images they categorized as appropriate for use. Similarly, overarching themes regarding what constituted an endorsed versus not-endorsed image were discussed with participants in real-time, and participants and the research team refined the themes followed by member-checking to confirm all findings with participants. Participants identified unanticipated themes. For example, participants emphasized that some images were triggering, not just negative. A category was added for images that made individuals reactive and think of using again (i.e., relapse). The research team identified common themes, which were used to inform recommendations. Through member checking all do/do not use recommendations and themes were confirmed by participants.

Member checking, or respondent validation, was a key component of the RQI process. Member checking builds trust and engagement with research participants. In addition to being provided immediate access to categories and themes during the virtual focus groups and interviews, participants were provided with drafts of written findings and recommendations over the course of several months following data collection. This process focused on checking for accuracy and ensuring recommendations reflected the experience and input of participants with lived experience with SUD.

## Results

Participant demographics are described in Table 1. Eight participants identified as female and six as male, four identified as Black, two as Native American, two identified as Hispanic/Latino, and one as more than one race (*Native American and White)*. The remaining five participants identified as White. The mean age was 44 years (range = 25—66). The 14 participants represented different types of SUD history: four with an opioid use disorder, three with a stimulant use disorder (methamphetamine or cocaine), three with alcohol use disorder, and four with polysubstance use disorder. All participants identified as being in recovery, with a range of time in recovery from a few weeks to 26 years. The majority of participants reported a history of justice-involvement (86%). The geographic breakdown of participant locations was as follows: three resided in Illinois, two in North Carolina, and one participant in each of the following states: Idaho, Oregon, Georgia, Florida, Kentucky, California, Minnesota, New York and Missouri. We describe themes that emerged from the analysis below of six distinct categories of imagery.

**Table 1:** Demographic characteristics (*n* = 14)

Characteristic	
Gender, n(%)	
Men	6 (42.9%)
Women	8 (57.1%)
Race/Ethnicity, n (%)	
More than one race, non-Hispanic	1 (7.14%)
Hispanic	2 (14.3%)
Native American, non-Hispanic	2 (14.3%)
Black, non-Hispanic	4 (28.6%)
White, non-Hispanic	5 (35.7%)
Age, Mean (SD)	44 (12.4)
Education, n (%)	
High School Degree	2 (14.3%)
Some College	2 (14.3%)
Associate’s Degree	3 (21.4%)
Bachelor’s Degree	3 (21.4%)
Master’s Degree	3 (21.4%)
Ph.D	1 (7.1%)
Type of Substance Use Disorder, n(%)	
Opioid Use Disorder	4 (33.3%)
Stimulant Use Disorder	3 (21.4%)
Alcohol Use Disorder	3 (21.4%)
Polysubstance Use Disorder	4 (33.3%)
Justice Involvement, n (%)	12 (85.7%)
Interview Type, n (%)	
Focus Group	8 (57.1%)
One-on-one Interview	6 (42.9%)

### Treatment and patient imagery

**Fig. 1:**
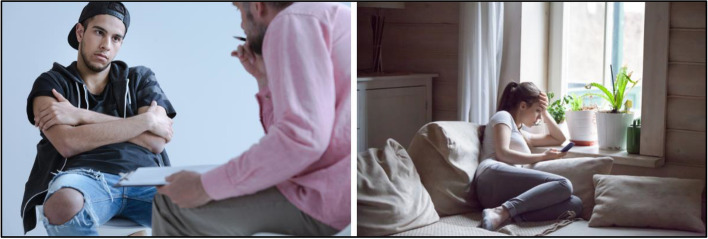
Stigmatizing treatment and patient imagery (Not Recommended to Use)

Nine images in the category of treatment and patient imagery were coded based on participant feedback. Participants identified two images as “do not use” due to negative representation and stigmatizing effects. Rejected photos include images of individuals who look like they are struggling in treatment or pictures that resemble private treatment websites, described by participants as overly dramatized and staged (Fig. 1). Imagery that conveys a lack of interest in treatment was reminiscent of coerced treatment for one participant. Several participants expressed concern that photos depicting patients struggling in treatment could reinforce isolation and despair, and potentially depict receiving treatment as a negative experience.

Poor treatment experiences were a salient subtheme in responses to treatment setting imagery. A 28-year-old female reported that the images led her to remember such an experience, she shared:

“I've had bad experiences at hospitals, to be honest. I have been abused by nursing staff, I have been abused by EMTs, I have been abused by doctors… you know, verbally abused, and I think that I've heard a lot of other people have those same experiences. For example, one of the times that I overdosed, the EMTs were just steadily making fun of me. And that's a crazy thing to wake up to in an ambulance. Like having somebody go through your backpack and, at the time I was homeless, so I mean for them to be going through my backpack and like ripping things out and saying, ‘Oh, this is disgusting,’ like ‘your backpack is so dirty,’ like, ‘you're a disgusting person…’ after I almost just died is kind of horrifying… and that stuff happens all the time and… I've had nurses that are so hateful. Like, it's very clear that they hate addicts and… want to make sure that I know that I'm just, like, the scum of the earth.”

**Fig. 2:**
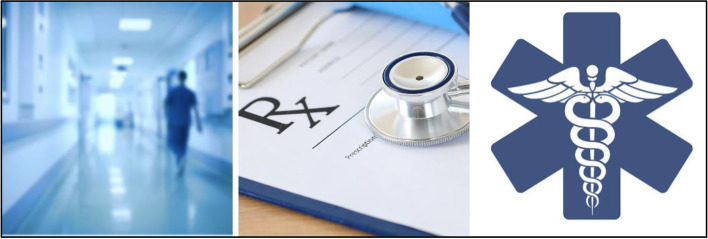
Non-stigmatizing medical imagery to use

Participants endorsed several medical images as “do use”, including photos of stethoscopes, medical icons, external and internal photos of a hospital without patients present, and prescription pads (Fig. 2).

For example, one participant emphasized support for photos that depicted a prescription pad or a prescription as a means to underscore the availability of medications to treat addiction, which she shared is still largely unknown among the patient population:

“A lot of people don't know that treatment [is there] other [than] going through withdrawal. I mean, because even I knew a girl like she was like ‘Oh, I have to lock myself in the basement for two weeks.’ I'm like, for what? And she was like ‘So I can, you know, detox.’ They don't know that there's other stuff out there other than you know just going cold turkey because that can really like traumatize you because you're like okay, I know I have to do this, I want to do this, but I have to do it in this painful way to get better and then nine times out of ten you're going to relapse. That's a really horrible feeling of going through withdrawal.”

**Fig. 3:**
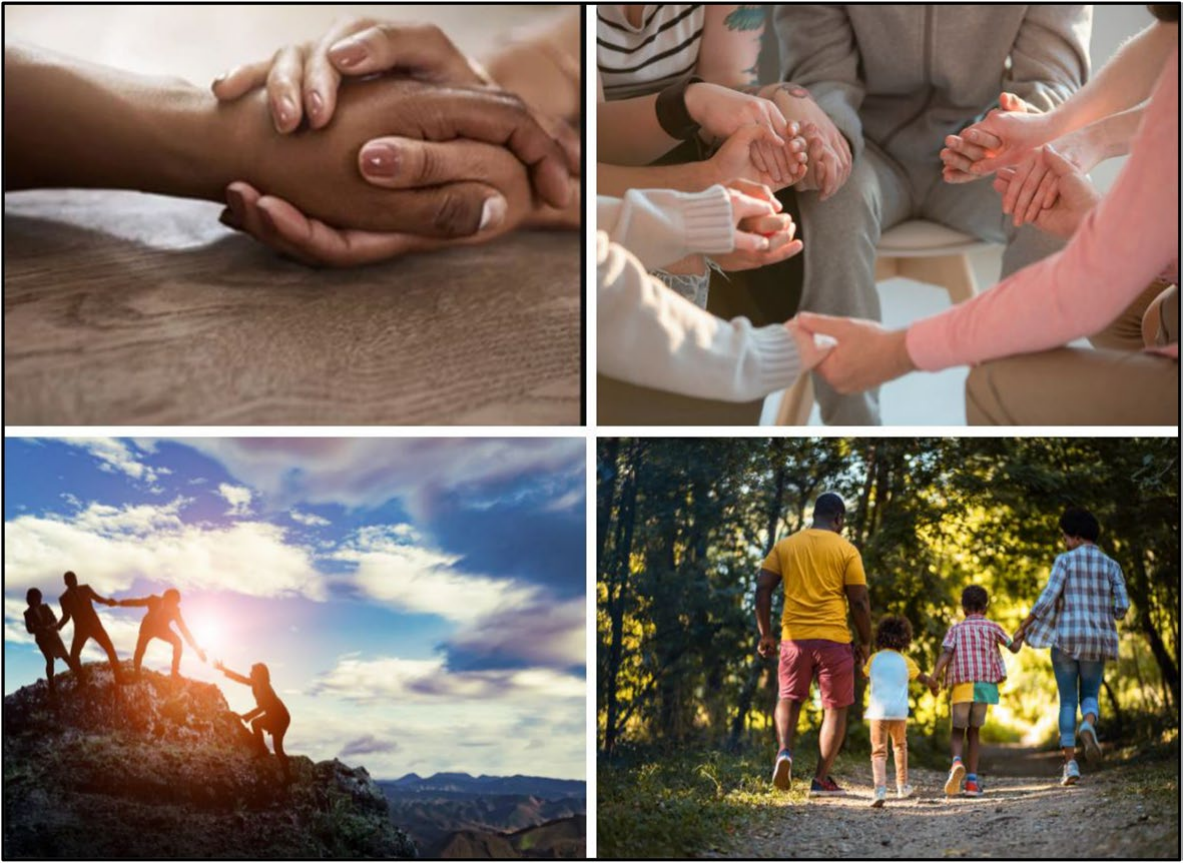
Non-stigmatizing treatment and patient imagery to use

Participants endorsed four images depicting treatment and patients, including photos of hand-holding, and individuals in a circle to illustrate group therapy or mutual aid support groups (Fig. 3). A study participant shared: “I love the hand holding, because I think it communicates if you're talking about patient services treatment it communicates what we actually do to help people get to where they need to be is that's very embracing.” Discussion centered around utilizing photos of wellness, health, and vitality when depicting individuals receiving treatment for substance use disorder or in recovery. Participants shared that images including nature and landscape felt positive and reinforced themes of new beginnings and health.

The most endorsed image among participants during the treatment and recovery section was an image of people helping to pull a peer up a hill with a landscape and a bright sky in the background, followed by a photo of a family holding hands, walking through a natural landscape (Fig. 3), all classified as “do use”. A study participant added:

“Recovery was about getting my life back, because there was stuff I didn't do because I was so focused on getting drugs and making sure I had what I needed to be able to function, to be able to be a mother. And then, you know, afterwards I am able to have the money to do stuff with my daughter and go to these places, and actually be able to have fun and not be like okay I'm about to run out, let me see who I can call or you know spending all my days doing other stuff. I mean to me it's like your way recovery is to get your life back. You're able to enjoy your family your friends and life itself. I love those photos.”

Participants again highlighted the importance of showing “all kinds” of diversity (e.g., gender, age) to be depicted in both patient and physician roles represented. In response to the image of medical personnel in the treatment/hospital setting one participant shared:

“I definitely agree with, like, showing racial diversity and age diversity and all kinds of gender diversity… and diversity among personnel is great, but I do think the patient should be shown… and have that same diversity applied to them because… the interaction between, like, medical staff and patients… that is a really big part of going to a hospital… I think that eliminating [patients], in a way, can have, like, the unintended effect of silencing those narratives that come from patient-personnel interactions.”

### Substance use disorder imagery

**Fig. 4:**
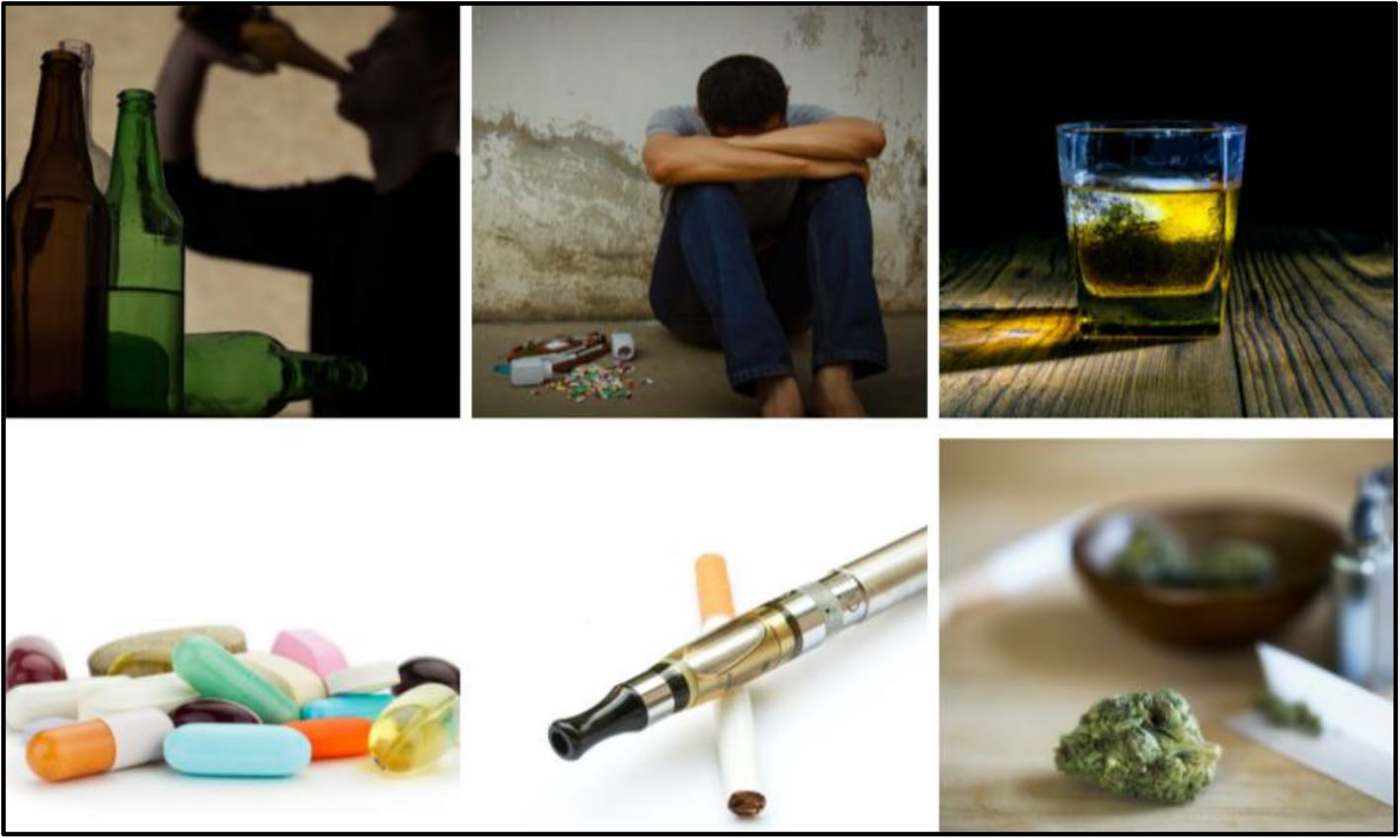
Stigmatizing SUD imagery (Not Recommended to Use)

Sixteen photos that depicted different types of substance use disorders were discussed during interviews and focus group sessions with participants. Realistic depictions of actual substances and alcohol, whether powders, crystals, marijuana, pills, alcohol containers or servings, or cigarettes/vapes were categorized as stigmatizing by participants (Fig. 4). Participants identified these images as “do not use” due to reinforcement of stigmatizing views of individuals with SUD. “It's a lot. It is very stigmatizing when it comes to what the images are saying,” shared one participant in recovery.

**Fig. 5:**
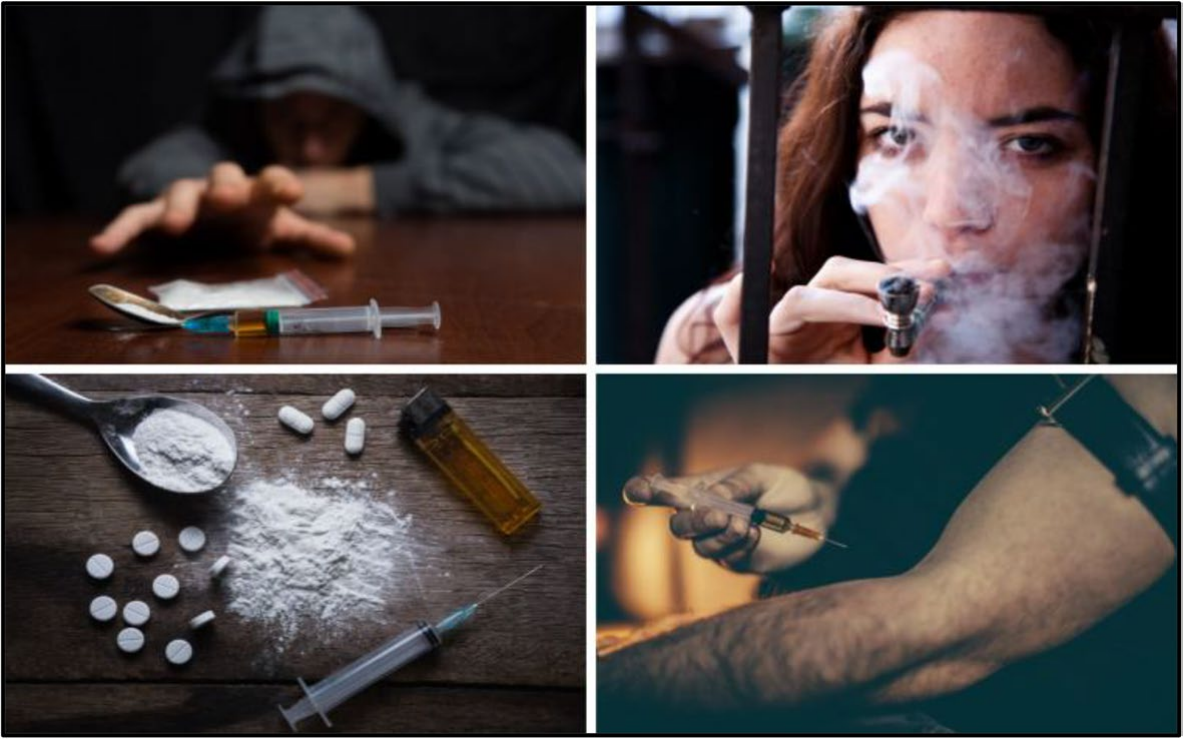
Stigmatizing and triggering SUD imagery (Not Recommended to Use)

Participants identified images of people using substances and paraphernalia (needles, syringes, spoons, or lighters) as both stigmatizing and triggering, with participants reporting that the images brought up urges or cravings to use substances (Fig. 5). These images were categorized as “do not use”. A participant reinforced during the interviews that this set of imagery is both stigmatizing and triggering:

“I could take, you know, any of those syringe ones into a crystal meth meeting and, you know, people with 10 years of sobriety would still be triggered by those images of someone shooting up. Or if you put a meth pipe there, for example, with smoke going out of it…it's, it's going to be triggering.”

Participants tended to categorize images featuring substances as triggering when they related to their own primary SUD diagnosis. A study participant whose SUD involved injecting drugs shared: “Yeah, I would just say, like, you know, obviously… the one with the dude tying off and shooting up is pretty, like, whoa… it’s out there… if it’s like showing the drink and the pills, I’m ok… but not if it’s showing the cocaine, and the heroin, and the meth… “

**Fig. 6:**
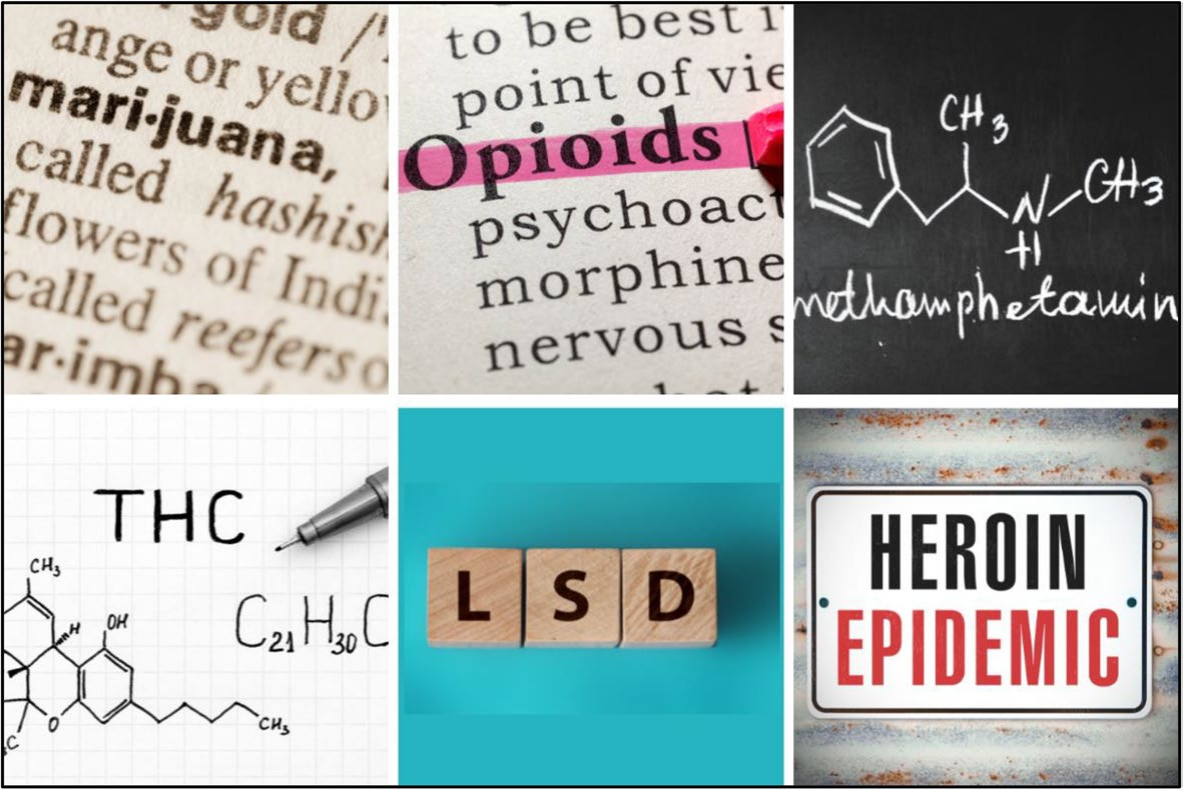
Non-stigmatizing SUD imagery to use

Participants endorsed using conceptual image options, such as molecular symbols of the SUD type, definitions of the type of substance from the dictionary, and typography options (Fig. 6) as “do use”. A participant shared that the conceptual imagery “keeps it scientific. It keeps it fact-based.”

### Criminal justice imagery

Focus group and interview participants responded to criminal justice imagery along with SUD imagery to identify characteristics of stigmatizing and non-stigmatizing images from the perspective of individuals in recovery from substance use.

#### Law enforcement

**Fig. 7:**
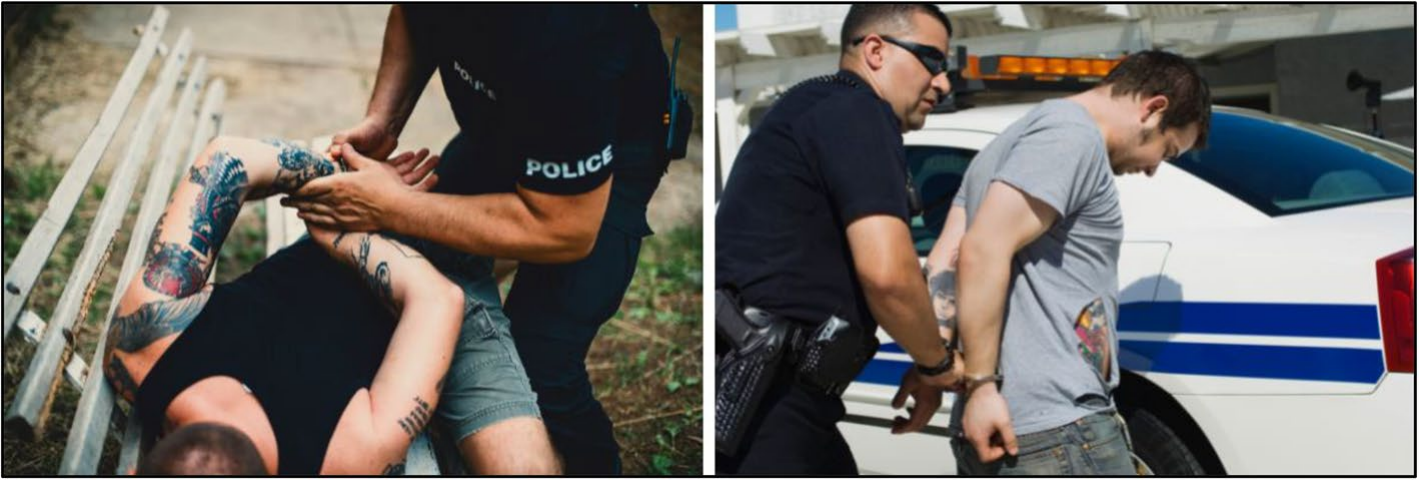
Stigmatizing law enforcement imagery (Do Not Use)

Four sample images related to law enforcement engagement were displayed for participant feedback. Statements and feedback from participants showed a negative reaction to depictions of individuals in handcuffs and being arrested (Fig. 7), which were categorized “do not use”. One participant shared: “I don't know if we call it PTSD, but I immediately looked at the guy being handcuffed and it felt violent. It felt aggressive.” Another participant also described the images portraying an arrest as “aggressive.” She added: “I think that that could be very traumatizing to somebody who's been put in that kind of a hold before.” While one participant explained: “I think they're negative photos for the police department, because it makes them look brutal.”

A study participant commented on the use of imagery of handcuffs or restraints: “Anyone in handcuffs–that's not their best day. It's not the right way to portray individuals going into the criminal justice system or with substance use disorders.”

A handful of participants added concerns about intersectionality and racism, responding to frequent images of White law enforcement officers arresting African American or Hispanic/Latino individuals. A participant shared: “A lot of these images that are used in this way don't show White people getting arrested they show people who are not white getting arrested by white officers.” They continued: “I think it’s traumatic that a lot of the images used in this way don’t show white people getting arrested.” Participants expressed the importance of equal representation across different races and ethnicities in images, from depictions of law enforcement or other positions of authority to images of those who are justice-involved or requiring SUD services. They also voiced concern about the stigmatizing representations of individuals with tattoos. One participant shared: “… yeah, well they're showing too much of what the guy looks like, but nothing about the officer, you know what I mean? And he's too identifiable by the tattoos. Anybody would know that knows him… that's just not right, you know?”.

**Fig. 8:**
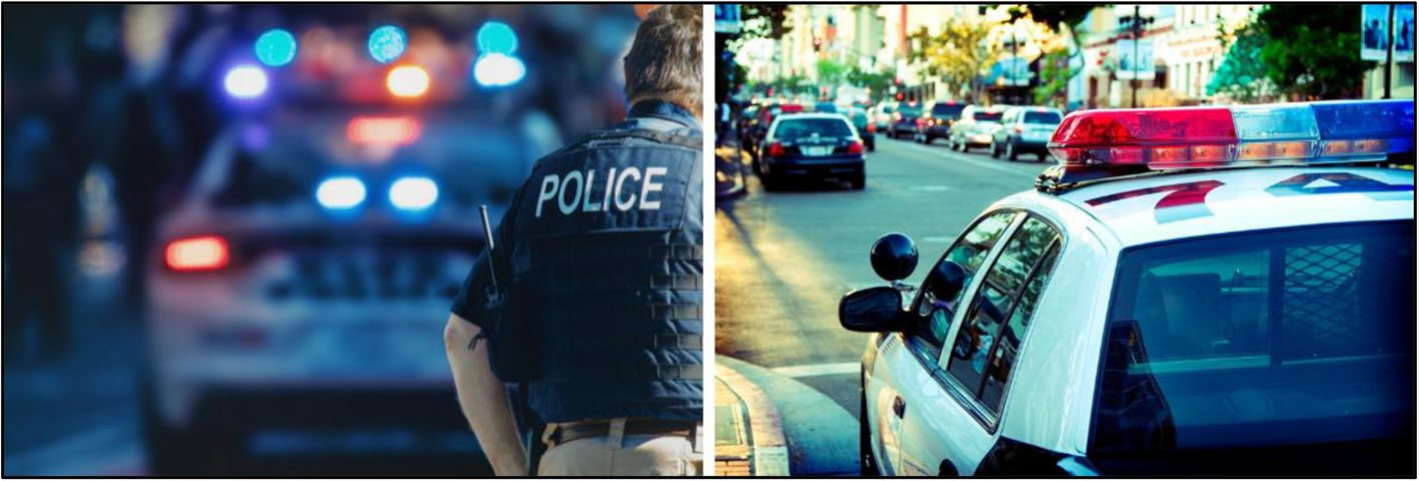
Non-stigmatizing law enforcement imagery to use

Participants endorsed photos of police cars, sirens, or police stations (Fig. 8) as non-stigmatizing. Participants agreed with police representation of officers alone (without an individual being arrested or depicted unfavorably) and helping in the community in pro-social activities. One participant shared their preference for images that show “interaction with the community in a positive photograph or something with a cop smiling, the person smiling, engaging, helping the old lady across the street that kind of thing.” Several participants shared that photos of officers or police cars reinforce safety and security. Another participant commented: “these images here make me feel safe.”

#### Courts

**Fig. 9:**
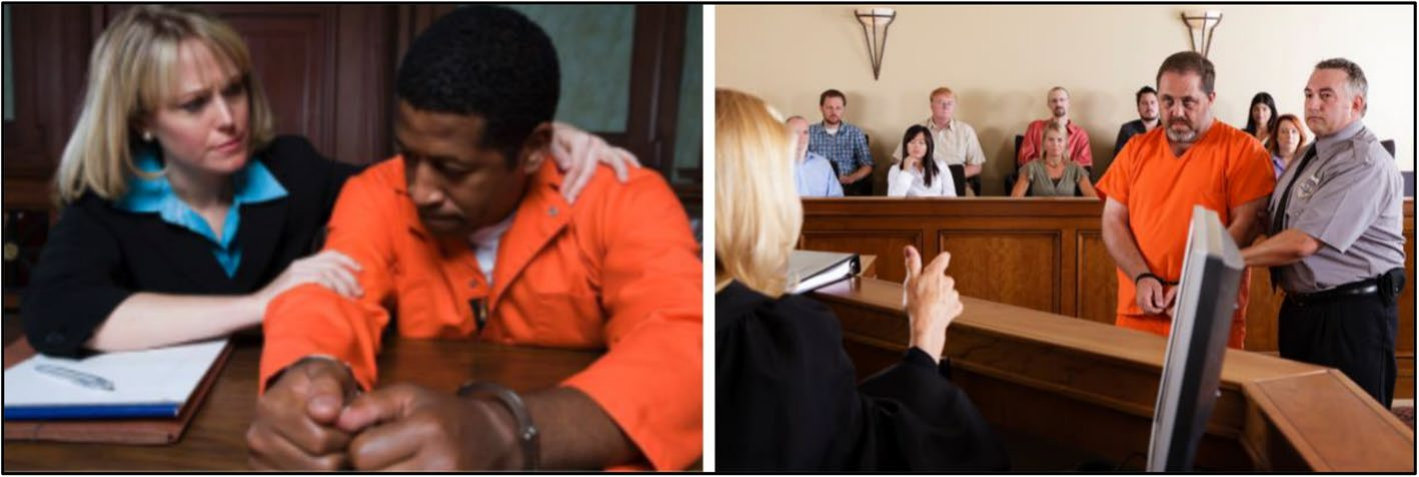
Stigmatizing courtroom imagery (Not Recommended to Use)

Four court-related images were shared with participants for feedback. Participants shared negative responses and emotional reactions to depictions of individuals in prison jumpsuits or handcuffs (Fig. 9), which were categorized as “do not use”. A research participant shared: “You know, I spent a lot of time in the orange suits…no I don't think that's a good one.” Another individual shared: “You definitely don't want Black people in the jumpsuits, you know what I mean? Like it's not a good look for us at all.” And another participant added: “It made me feel nervous and nauseous.” Discussions demonstrated concerns that jumpsuit and handcuffs suggest guilt regardless of the status of the individual. “It's just, you know, that orange jumpsuit. It's just, again, kind of this presumption of I'm guilty until proven innocent almost instead of innocent until proven guilty.” Another individual shared: "The pictures and the imagery send a whole other negative message and triggers a lot of emotions around unfair sentencing and unfair representation."

Another study participant provided a gender-specific characterization of the requirement of jumpsuits in courtrooms and other settings:

“I think they should allow us to go to court in our own clothes. I think that orange jumpsuits are very embarrassing. They are usually stained... they give us one jumpsuit a week, you know, and they don't wash them. They don't use detergent on them; they only use water at the jail. Women, you know, have their menstrual cycles and they put them back out there, like that you know with stains.”

**Fig. 10:**
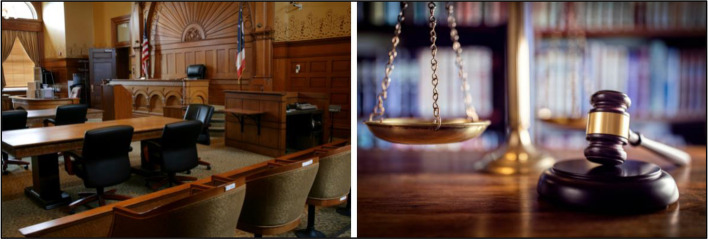
Non-stigmatizing courtroom imagery to use

In contrast, participants endorsed photos of empty courtrooms, a gavel, or the scales of justice to convey material or information related to the judicial system (Fig. 10), which were categorized as “do use”. If imagery of people is required, participants expressed preference for images of a judge, district attorney or other professional without defendants in the frame. One participant commented: “I think this picture, these pictures right here, especially one with a scale and the one is a gavel, I think is a good depiction of the justice system or court.” Another added that photos of a gavel “represents justice.” Another individual in recovery shared: “I think, keeping it focused on the court system and the stakeholders in that system, which are the judges, court reporters.”

### Jails and prisons

**Fig. 11:**
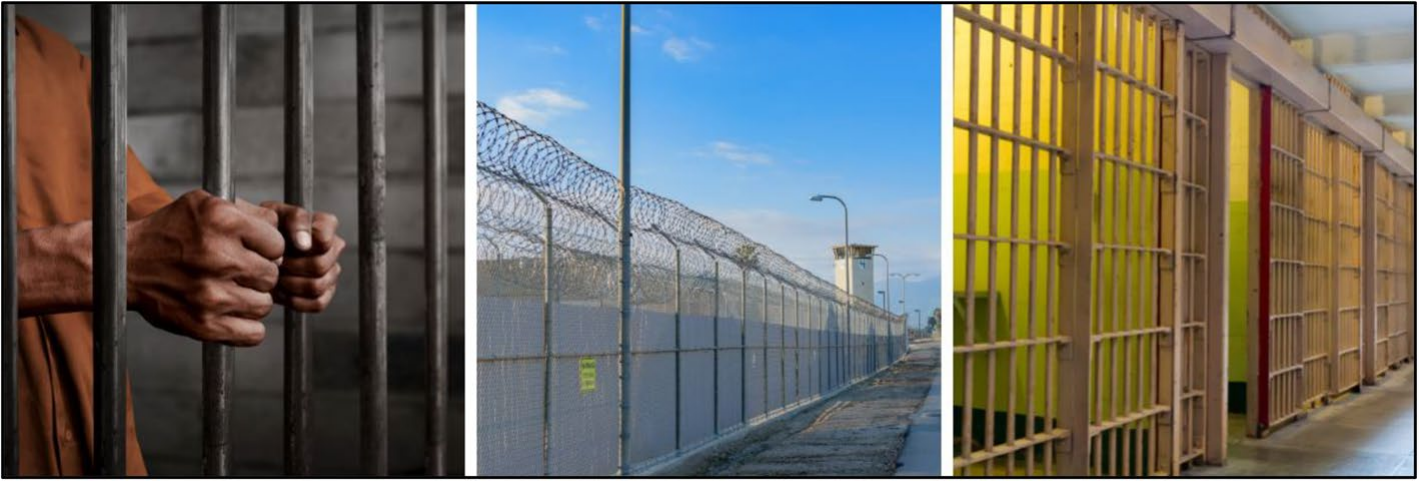
Stigmatizing prison and jail imagery (Not Recommended to Use)

For carceral setting imagery images of bars, rows of cells, individuals behind bars, and images of prison or jail structures that include barbed wire or gun towers were rejected by participants (Fig. 11), categorized as “do not use.” Several images were identified by participants as traumatizing, cueing negative and trauma-inducing memories. A study participant shared: “There's just something really brutal about the rows and rows [of cells]; it's pretty dehumanizing. I mean, the cell itself -- like putting a person in a cage -- is already dehumanizing enough." Another participant shared: “We didn't have gun towers in our prison.. yeah that's intimidating.”

**Fig. 12:**
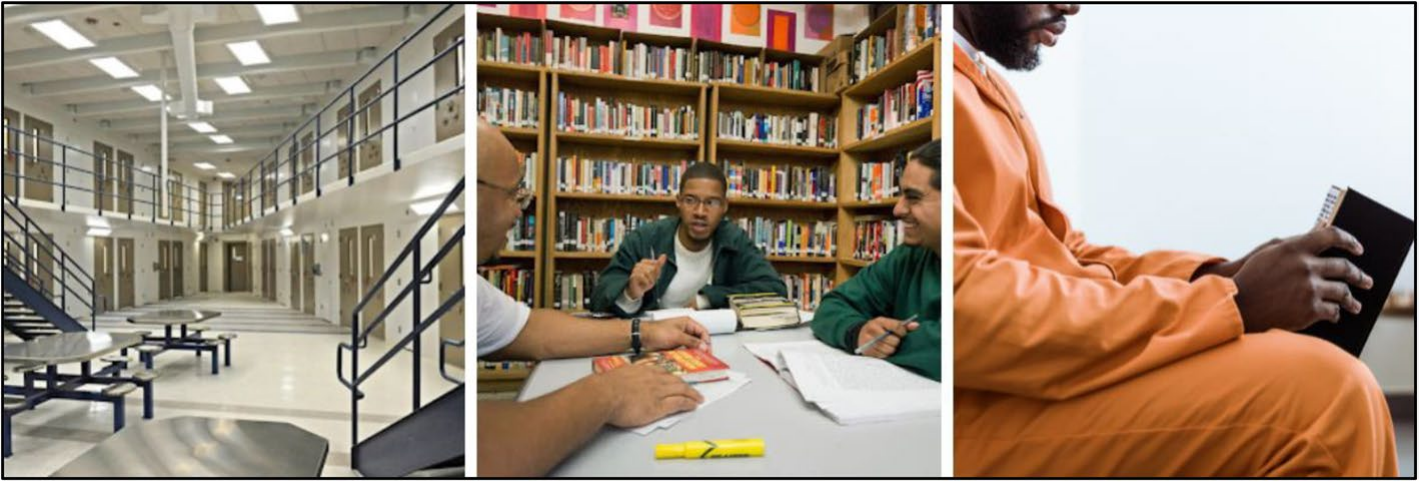
Non-stigmatizing prison and jail imagery to use

Participants identified more acceptable imagery for individuals with lived experience to convey prison contexts, recommending images that focus on positive activities during incarceration and photos of modern, updated common areas, cafeterias, prison libraries, and individuals in classrooms and vocational trainings to indicate prison settings (Fig. 12), which were categorized as “do use.” To ensure accuracy in replacement images, member checking was performed via email to share revised images that reflected their recommendations. Participants were asked to verify whether the images accurately represented their feedback, which was confirmed.

“Pictures of the prison library or something ...or recreation, or something like that, where it's like showing helpful things you know what I mean. Where it's more of an opportunity for growth than being caged up or punished, which we all know, it's punishing to lose your freedom but it's supposed to be so that you can change your behavior.”

### Reentry, probation and parole

**Fig. 13:**
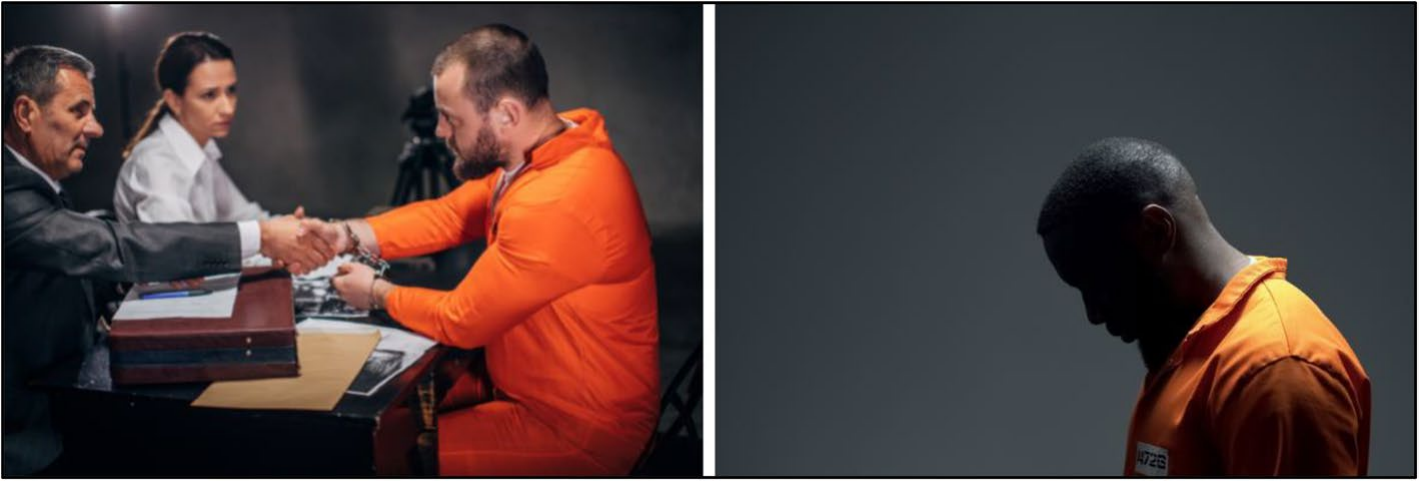
Stigmatizing reentry imagery (Not Recommended to Use)

The broad scope of services and personnel involved in probation, parole and reentry can cover a variety of images from transition into the community to case management and participant engagement. Seven images were discussed to convey this context. Participants expressed negative emotional reactions to images of individuals still in prison jumpsuits or handcuffs in these settings, or any image that reflects despair (Fig. 13).

“You have a dude in an orange jumpsuit. He is getting ready to be released, but they still got him all cuffed up. This is about reentry, he shouldn't be handcuffed he should be talking to a prospective employer or something or you know what I mean, he should be like getting a diploma for finishing a program that he did in jail or whatever, and you know.”

In a similar vein, another participant shared: “So much shame and no background, he almost just blends into the background it's like yeah there's no hope there. Oh God, there's no hope.”

**Fig. 14:**
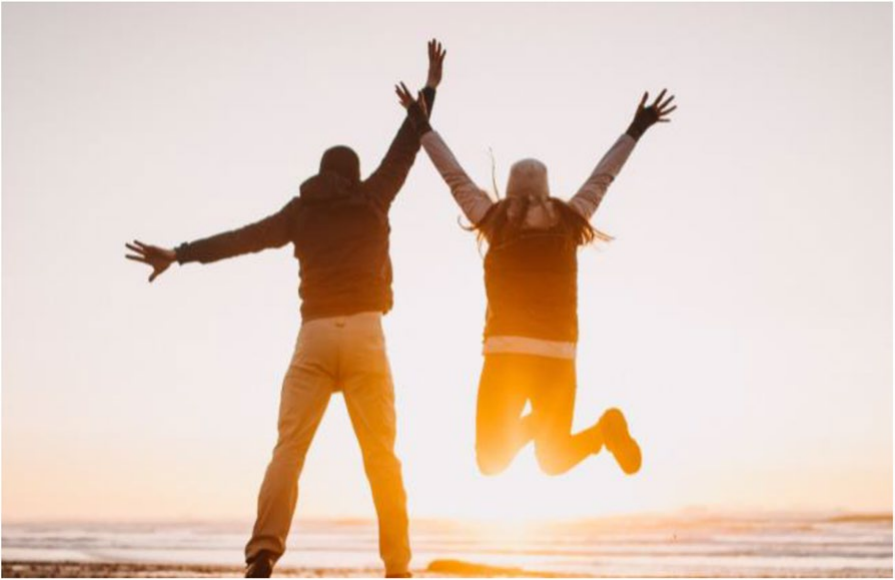
Unrealistic reentry imagery to not use

Unrealistic photos and descriptions of reentry and supervision also elicited negative feedback from participants, such as individuals jumping for joy or cheering (Fig. 14). Participants rejected the imagery as unrealistic to the actual experience of reentry and community supervision. One individual shared: “I don't think anybody's like that happy to be on parole, you know? I mean like, yeah, they're getting out so they could be like jumping for joy, but I don't think that's very accurate depiction [of what it’s] like.”

**Fig. 15:**
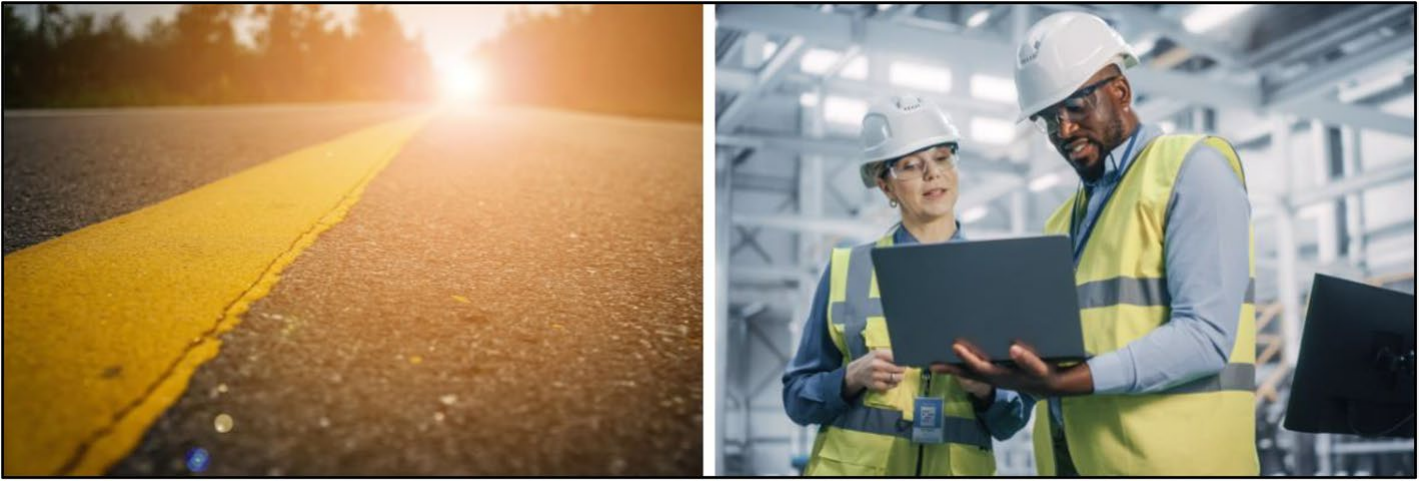
Non-stigmatizing reentry imagery to use

Photos of roads and pathways with hopeful coloring, doors opening with a hopeful theme, abstract images of the community, and individuals working within the community received positive feedback from participants in conveying reentry (Fig. 15). “I like the one with the road on the road with like the sunshine down the road you know I mean like there's light at the end of the road.”

Similarly, another participant shared the following in response to the reentry imagery:

“I think all images for reentry and parole definitely should have brightness… it helps to shine a light on the process [of reentry and parole]... I really like the imagery of walking from the darkness into the light as a transition of reentry and, you know… from hopelessness to hope… that's what reentry should actually signal to people… coming out of the dark.”

Discussion demonstrated participants prefer aspirational, hopeful images to represent the promise of reentry and community reintegration. Participants suggested more community activity photos and employment-focused imagery. One individual shared: “I would show more community pictures… healthy community pictures. Something in a park, for instance, where there’s kids playing and families socializing… it might just be at a baseball game or a football game or any kind of sports event… the things that people do in community… it could be church, you know. It could even be a recovery meeting.”

While there was broad consensus on image recommendations for the “do and don’t use” framework across the six categories of imagery, some discrepancies were found. Two study participants, one in law enforcement and another in the research field, had different responses to a range of imagery than other study respondents, possibly relating to professional experience. For example, the study participant in law enforcement endorsed all law enforcement imagery, including photos rejected by other participants. Also encountered were discrepancies in how people react to specific images. For example, four participants responded negatively to an image with a clinician and patient where the clinician was holding a clipboard and taking notes. Participants shared that the image felt “invasive,” was reminiscent of probation and other experiences where data is collected, and expressed concerns about not knowing what clinical or supervising professionals are writing or reporting about an individual.

## Discussion

This is one of the few studies to date that has sought to understand stigmatizing imagery through the feedback of individuals with lived substance use disorder experience. Stigma is a major barrier to treatment and recovery for individuals with SUD, with intersecting stigma experiences for those who are also justice-involved. Results identified imagery of substance use and criminal justice contact that is stigmatizing, along with alternate images categorized as non-stigmatizing by study participants.

Our findings have implications for the recommended depiction of addiction by the media, research publications, policymakers and other institutions. The recommendations can provide the basis for guidance surrounding non-stigmatizing imagery to use in reference to SUD. An outline of recommended imagery to use, and to avoid, informed by research participants in this study is provided in Table 2.

**Table 2:** Imagery-induced stigma by setting

Category	Non-Stigmatizing Imagery	Stigmatizing Imagery
Treatment/Patients	Holding hands, group therapy, support groups, people helping to support a peer Stethoscopes, medical icons, external and internal photos of a hospital without patients present, prescription pads, and doctors without patients in frame with racial diversity	Distressed or unhappy individuals, overly-dramatized photos
Types of SUD	Molecular symbols of the SUD type, definitions from the dictionary, and typography	Images of drugs, alcohol, pills, and paraphernalia; images of individuals using or preparing substances
Law Enforcement	Images with police cars, sirens, police stations, and police officers without other individuals in the image Images with equal representation across different races and ethnicities in images, from depictions of law enforcement or other positions of authority to images of those who are justice-involved or requiring SUD services Images where the representation of officers are alone (without an individual being arrested or depicted unfavorably) and helping in the community in pro-social activities	Images of people being arrested or in handcuffs Images of White law enforcement officers and those being arrested represented by African American or Hispanic/Latino individuals
Courts	Photos of empty courtrooms, a gavel, or the scales of justice to convey material or information related to the judicial system	Depictions of individuals in prison jumpsuits or handcuffs
Jail/Prison	Images that focus on positive activities during incarceration and photos of modern, updated common areas, cafeterias, prison libraries, and individuals in classrooms and vocational trainings to indicate prison settings	Images of bars, rows of cells, individuals behind bars, and images of prison or jail structures that include barbed wire or gun towers
Reentry	Photos of roads and pathways with hopeful coloring, doors opening with a hopeful theme and abstract images of the community Aspirational, hopeful images to represent the promise of reentry and community reintegration. Participants suggested more community activity photos and employment-focused imagery	Images of individuals still in prison jumpsuits or handcuffs in these settings, or any image that reflects despair Unrealistic photos and descriptions of reentry and supervision

While the study’s primary goal was to inform practical guidance on the representation of SUD in various formats to reduce stigma, the qualitative feedback was also useful for identifying acute areas of concern for patients. For example, the sample of participants emphasized the importance of diversity in race/ethnicity, gender and age for representations of both individuals with substance use disorders or justice involvement and clinicians in imagery. The importance of ensuring imagery about SUD does not perpetuate stereotypes such as dangerousness or prejudice like fear or lack of trust. Stigmatizing images can create additional barriers and challenges for individuals with SUD, from social distance to discrimination. Another key concern that emerged was that images depicting treatment be inclusive and hopeful, not depressing or negative which may have the unintended consequence of dissuading individuals from seeking help.

The representation of types of SUD was a significant concern from study participants. Much of the imagery currently used to identify different types of substance use disorders (opioid use disorder, stimulant use disorder, alcohol use disorder) was identified as both triggering and stigmatizing by those in recovery from SUD. The unanticipated concept of imagery-induced triggering and cue reactivity emerged in the interviews and may suggest it is inappropriate to use drug use and drug paraphernalia imagery to depict substance use or misuse. These findings are consistent with research on cue-reactivity in behavioral addictions, which finds that various cues become associated with the rewarding properties of the substance for individuals with a SUD (Carter & Tiffany, 1999). Cue reactivity is considered a risk factor for relapse for individuals in recovery.

It is important to mention that not every participant reacted to images the same. Even if the guidance outlined in this report is followed, some individuals may react negatively to specific images. Individual’s past experiences, positive or negative, impact how they view and assign meaning to each image. For example, one image that not all participants agreed on was having a clipboard within a photo representing treatment of a patient.

### Strengths, limitations and future directions

Researchers and participants met through Zoom to conduct the study due to COVID-19, which allowed for real time answers and member checking. The study utilized a rapid qualitative inquiry where a team-based framework was implemented where individuals were asked if they agreed with the do’s and don’ts that were outlined for each image then participants provided feedback on what their recommendations and improvements would be for each category. This was an iterative process that allowed researchers to code in the moment and analyze the data as participants shared. The process facilitated the search for images that participants were describing and present them to the group. Through member checking and back and forth dialogue recommendations were finalized and researchers were able to check with participants if they approved of the images that were found. This two-pronged approach was one of the strengths of the study.

While this study addressed a gap in research and offers recommendations regarding non-stigmatizing imagery to use in substance use disorder contexts, it is not without limits. This is an understudied area of research, therefore, RQI provided utility and was appropriate for the goal of the study, which was to elicit recommendations for non-stigmatizing imagery from individuals with lived experience. However, this study is just the first step. More research is needed to peel back the layers of why certain images were deemed stigmatizing and to test whether these findings remain valid across different populations.

The study sample only included individuals who could speak English; this potentially creates a bias since the lived experiences of non-English speaking individuals with justice involvement and substance use disorder may differ from their English-speaking counterparts. Furthermore, Hispanic individuals are the fastest growing subgroup of prisoners in the United States and currently comprise 30.2% of the U.S. prison population (Federal Bureau of Prisons, 2022). While the current study provides a starting place for understanding stigmatizing imagery in SUD and justice-involved contexts, future research should prioritize the inclusion of non-English speaking participants to test the current study’s findings and provide an intersectional viewpoint of this understudied area of research.

Finally, participants were recruited from APF’s advisory board, patient and stakeholder network, as well as from their network of individuals who receive clinical services or who participate in APF’s recovery programming. It is possible that these individuals may have had a greater vested interest in this topic than others. This study is nonetheless a contribution in that it provides concrete recommendations for non-stigmatizing imagery, informed by individuals with lived experiences with substance use disorder and with the justice system.

## Conclusion

Stigmatizing imagery can reinforce negative attitudes and beliefs about individuals with substance use disorders, which create barriers to care. The current study can be helpful in informing imagery used to depict addiction, individuals with substance use disorder, and individuals involved in the justice system for various fields. In particular, qualitative feedback from patients on triggering effects and reactivity to visual cues suggests it is never appropriate to use drug use and drug paraphernalia imagery to depict substance use or misuse. Future research is needed to further elucidate these themes and provide an in-depth understanding of the different categories of SUD imagery (non-stigmatizing, stigmatizing and triggering). Findings from the current study suggest that qualitative feedback and collaboration with individuals with lived experience can inform how we represent addiction and interactions with the criminal legal system in various forms of media to reduce stigma.

## Data Availability

The data that support the findings of this study are available on request from the corresponding author, JH. The data are not publicly available due to identifying information that could compromise research participant privacy and/or consent.

## References

[CR1] American Medical Association (2021). How language makes a difference in treating substance-use disorder.

[CR2] Ashford RD, Brown AM, Curtis B (2018). Substance use recovery and linguistics: The impact of word choice on explicit and implicit bias. Drug and Alcohol Dependence.

[CR3] Associated Press (2017). Data journalism chapter debuts in 2017 AP Stylebook.

[CR4] Beebe J (2014). Rapid qualitative inquiry: A field guide to team-based assessment.

[CR5] Carter BL, Tiffany ST (1999). Meta-analysis of cue-reactivity in addiction research. Addiction.

[CR6] Corrigan PW, Watson AC, Barr L (2006). The self–stigma of mental illness: Implications for self–esteem and self–efficacy. Journal of Social and Clinical Psychology.

[CR7] Daundasekara SS, Arlinghaus KR, Johnston CA (2019). The importance of language in behavior change. American Journal of Lifestyle Medicine.

[CR8] Earnshaw VA (2020). Stigma and substance use disorders: A clinical, research, and advocacy agenda. American Psychologist.

[CR9] Federal Bureau of Prisons (2022). BOP statistics: Inmate ethnicity.

[CR10] Goodyear K, Haass-koffler CL, Chavanne D (2018). Opioid use and stigma: the role of gender, language and precipitating events. Drug and Alcohol Dependence.

[CR11] International Society of Addiction Journal (2015). Statements and Guidelines ​Addiction Terminology.

[CR12] Kelly JF, Westerhoff CM (2010). Does it matter how we refer to individuals with substance-related conditions? A randomized study of two commonly used terms. International Journal of Drug Policy.

[CR13] Ledford, V., Lim, J. R., Namkoong, K., Chen, J., & Qin, Y. (2021). The influence of stigmatizing messages on danger appraisal: examining the model of stigma communication for opioid-related stigma, policy support, and related outcomes. *Health Communication*, 1–13. 10.1080/10410236.2021.1920710 10.1080/10410236.2021.1920710 33941010

[CR14] Moore KE, Tangney J (2017). Managing the concealable stigma of criminal justice system involvement: A longitudinal examination of anticipated stigma, social withdrawal, and post–release adjustment. Journal of Social Issues.

[CR15] Reed SK (2013). Thinking visually. Psychology Press.

[CR16] Stangl, A. L., Earnshaw, V. A., Logie, C. H., Van brakel, W., C. Simbayi, L., Barré, I., & Dovidio, J. F. (2019). The health stigma and discrimination framework: a global, crosscutting framework to inform research, intervention development, and policy on health-related stigmas. *BMC Medicine*, *17*(1). 10.1186/s12916-019-1271-3 10.1186/s12916-019-1271-3 PMC6376797 30764826

[CR17] Substance Abuse and Mental Health Services Administration HHS (2015). National Survey on
Drug Use and Health 2015.

[CR18] Tsai AC, Kiang MV, Barnett ML, Beletsky L, Keyes KM, Mcginty EE, Smith LR, Strathdee SA, Wakeman SE, Venkataramani AS (2019). Stigma as a fundamental hindrance to the United States opioid overdose crisis response. PLOS Medicine.

[CR19] Turney K, Lee H, Comfort M (2013). Discrimination and psychological distress among recently released male prisoners. American Journal of Men’s Health.

